# Exosomes as a New Delivery Vehicle in Inflammatory Bowel Disease

**DOI:** 10.3390/pharmaceutics13101644

**Published:** 2021-10-09

**Authors:** Xiaomei Wang, Guoliang Zhou, Wanwan Zhou, Xin Wang, Xiao Wang, Chenggui Miao

**Affiliations:** 1 Department of Humanistic Nursing, School of Nursing, Anhui University of Chinese Medicine, Hefei 230012, China; wangxiaomei@ahtcm.edu.cn; 2 Department of Pharmacy, School of Life and Health Sciences, Anhui University of Science and Technology, Fengyang 233100, China; zhougl@ahstu.edu.cn; 3 Department of Pharmacology, School of Integrated Chinese and Western Medicine, Anhui University of Chinese Medicine, Hefei 230012, China; 2020204223003@stu.ahtcm.edu.cn (W.Z.); xinwang2019@stu.ahtcm.edu.cn (X.W.); 4 Department of Clinical Nursing, School of Nursing, Anhui University of Chinese Medicine, Hefei 230012, China; wangxiao@ahtcm.edu.cn; 5 Institute of Prevention and Treatment of Rheumatoid Arthritis of Chinese Medicine, Anhui University of Chinese Medicine, Hefei 230031, China

**Keywords:** inflammatory bowel disease, exosome, non-coding RNA, chronic relapsing inflammatory disease, delivery system

## Abstract

Inflammatory bowel disease (IBD) is a type of chronic relapsing inflammatory disease. The pathogenesis of IBD is still unclear, which may involve environmental factors, genetic factors, intestinal microbiota disorder, and abnormal immune responses. Exosomes (30–150 nm) are found in various body fluids, including blood, saliva, urine, and cerebrospinal fluid. Exosomes mediate intercellular communication and regulate cell biological activity by carrying non-coding RNAs, proteins, and lipids. There is evidence that exosomes are involved in the pathogenesis of IBD. In view of the important roles of exosomes in the pathogenesis of IBD, this work systematically reviews the latest research progress of exosomes in IBD, especially the roles of exosomes as non-coding RNA delivery systems in the pathogenesis of IBD, including a disordered immune response, barrier function, and intestinal microbiota. The review will help to clarify the pathogenesis of IBD and explore new diagnostic markers and therapeutic targets for patients with IBD.

## 1. Introduction

Inflammatory bowel disease (IBD) is a chronic relapsing inflammatory disease that can affect any part of the gastrointestinal tract. IBD includes two main diseases: Crohn’s disease (CD) and ulcerative colitis (UC). Although CD and UC usually have similar clinical manifestations, they affect different parts of the gastrointestinal tract, and the degree of intestinal wall inflammation is different. IBD is a multifactorial disease, which originates from the influence of environmental and genetic factors on the intestinal microbiome [1]. Pathological mechanism studies show that IBD seems to originate from an uncontrollable continuous inflammatory process, which often targets the intestinal microbiome of a genetically susceptible individual [2].

An abnormal intestinal mucosal immune system is an important inducer of IBD. IBD may be caused by the interaction of multiple factors, including environmental, genetic factors, intestinal microbiota, and immune response. It is known that the inflammatory response caused by the abnormal response of the intestinal mucosal immune system mediates the pathogenesis of IBD. For example, when the body produces abnormal immune responses to bacteria, viruses, or food particles, it triggers an intestinal inflammatory response and IBD [3]. Studies have shown that the intestinal microbiota is involved in the treatment of IBD. For example, transplantation of fecal microbiota from healthy controls into the intestines of IBD patients has been used clinically as an emerging treatment for IBD [4].

IBD genetics has also received extensive attention. IBD can be regarded as a complex genetic disease. Functional research inspired by IBD genetics has helped reveal the basic mechanisms of the interaction of the immune and host microbiome and has provided operable insights for therapeutic innovation [5]. IBD is regarded as a model disease, which reflects the complexity of coordinating the interaction among genetic, immune, and environmental variables affecting the disease. It is caused and amplified by a variety of genetic and environmental variables interfering with the immune microbiome [6].

IBD symptoms can be manifested as the intermittent recurrence and remission of static inflammation. UC affects the colon, and CD affects any region of the gastrointestinal tract but mainly the terminal ileum of the small intestine. Although IBD can occur at any age, IBD usually occurs in early adulthood, and the incidence rate is increasing in all ages. IBD has always been considered a western disease, but the incidence rate in the Eastern hemisphere is rapidly increasing—the following patterns of industrialization or westernization [7]. Clinical manifestations include diarrhea, abdominal pain, and bloody stool, and even various systemic complications, such as blurred vision, joint pain, rash, etc. The UC is a continuous inflammation of the colonic mucosa and submucosa. The disease usually involves the rectum first and gradually spreads to the whole colon. The CD can involve the whole digestive tract, which is a discontinuous full-thickness inflammation, and the most common involved parts are the terminal ileum, colon, and perianal [8].

Because there are many pathogenic factors and complex etiologies, the pathogenesis of IBD has not been fully clarified. The incidence rate of CD and UC in 100,000 patients in different regions of the world is 0.1–11 and 0.5–24.5, respectively, and the incidence rate of IBD is increasing [9]. Patients with severe symptoms suffer asphyxia due to severe complications. It has gradually become a common disease in the digestive department, and severe symptoms bring a heavy financial burden to families and society [10].

Diet and maintaining good nutritional status are very important for the recovery of patients with IBD. The intestines of IBD patients are usually difficult to absorb carbohydrates, proteins, fats, vitamins, and many trace elements. Furthermore, intestinal inflammation or drugs cause poor appetite, resulting in IBD patients often accompanied by varying degrees of malnutrition, which can affect the normal growth of children [11]. Nutritional support plays an important role in alleviating IBD symptoms and promoting healing. Maintaining good nutritional status is an important part of the treatment of IBD. There is no special diet for patients with IBD, and different foods can be tolerated by different people. Therefore, the general dietary principle of patients is to ensure their own dietary balance and avoid those foods that worsen their condition [12].

Exosomes are small membrane vesicles containing nucleic acids, proteins, and lipids. Many cell types can secrete exosomes under normal and pathological conditions. Exosomes mainly come from the multivesicle formed by the invagination of lysosomal particles in cells, which are released into the extracellular matrix after fusion between the multivesicle outer membrane and the cell membrane [13].

Exosomes naturally exist in various body fluids, including blood, saliva, urine, cerebrospinal fluid, and milk. The precise molecular mechanisms of their secretion, uptake, composition, and corresponding functions have become a new research interest [14]. Exosomes are currently regarded as membrane vesicles specifically secreted and participate in intercellular communication. There is a growing interest in the study of exosomes, whether it is studying their functions or understanding how they can be used in the development of minimally invasive diagnoses. Intestinal microbiota-derived exosomes deserve further study because they may reveal the potential mechanisms of the treatment of fecal microbiota transplantation [15].

**Figure 1:**
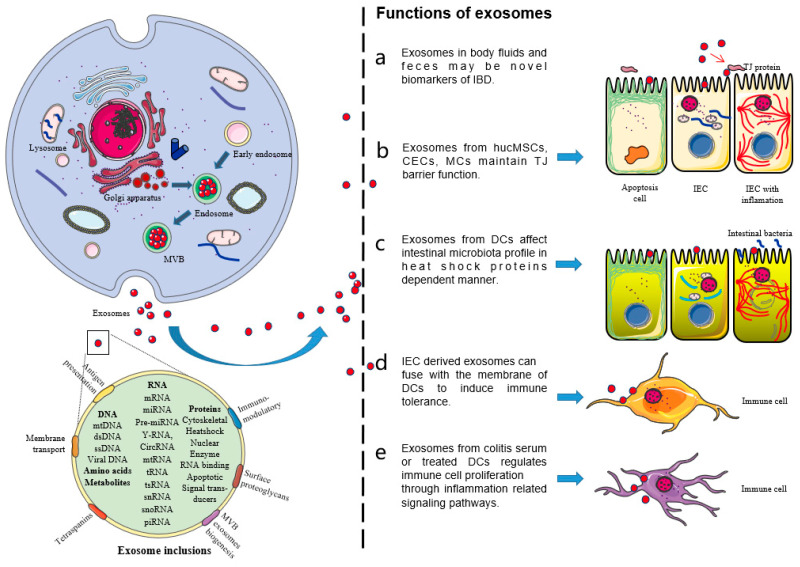
The maturation and secretion mechanisms of exosomes and the functions of exosomes in diseases. After most endosomes mature to multivesicular bodies (MVB) or late endosomes, their contents, RNAs, proteins, lipids, etc., are packaged as intraluminal vesicles (ILVs) in MVB. With the fusion of MVB and cell membrane, ILVs are released as exosomes and enter target cells by endocytosis, which affects the physiological and pathological mechanisms of target cells, such as proliferation, apoptosis, activation, and inhibition. Exosomes affect the functions of the target cells through their inclusions, such as non-coding RNA, proteins, and lipids. (**a**) Exosomes in body fluids and feces may be novel biomarkers of IBD. (**b**) Exosomes from human umbilical cord mesenchymal stem cells (hucMSCs), colonic epithelial cells (CECs), and mast cells (MCs) maintain the tight junction (TJ) barrier function. (**c**) Exosomes from dendritic cells (DCs) affect the intestinal microbiota profile in heat shock proteins-dependent manner. (**d**) Intestinal epithelial cell (IEC)-derived exosomes can fuse with the DCs membrane to induce immune tolerance. (**e**) Exosomes from colitis serum or treated DCs regulate the immune cell proliferation through inflammation-related signaling pathways.

Exosomes mediate intercellular communication by carrying nucleic acids, proteins, and lipids, which affect the pathogenesis of human diseases [16]. There is evidence that exosomes are involved in the pathogenesis of IBD [17] (Figure 1). In view of the important roles of exosomes in IBD, this paper systematically reviews the latest research progress on the roles of exosomes in IBD, especially the roles of exosomes as a non-coding RNA delivery system in the pathogenesis of IBD. This review will help to clarify the pathogenesis of IBD and explore new diagnostic markers and therapeutic targets for patients with IBD.

## 2. Exosomes in Body Fluids and Feces as Diagnostic and Therapeutic Targets for IBD

### 2.1. Serum Exosomes

#### 2.1.1. Serum Exosomes Affect the Effect of Vedolizumab (VDZ)

VDZ is a therapeutic monoclonal antibody approved for the treatment of IBD [18]. Exosomes express α4β7 integrin and compete with CD4^+^ T cells for binding to VDZ, resulting in a decrease in the number of VDZ bound to T cells. The target α4β7 integrin of VDZ is expressed on the surface of serum exosomes isolated from patients with UC. According to the expression difference of α4β7 integrin on the surface of exosomes, the exosome isolation effect against CD4^+^ T cells is increased in anti-TNF-exposed patients compared with anti-TNF-α-naïve patients. Exosomes bind to VDZ and, therefore, cannot block the mucosal addressin-cell adhesion molecule 1 (MAdCAM-1)-mediated lymphocyte adhesion. Previous treatment of patients with IBD changes the isolation ability of these circulating exosomes, thus reducing the efficacy of VDZ in patients with failed anti-TNF drug treatment. This also indirectly proves the important role of circulating exosomes in the pathogenesis of IBD [19,20].

#### 2.1.2. Pregnancy Zone Protein (PZP) in Serum Exosomes

PZP in serum exosomes is involved in the pathogenesis of IBD. Shao et al. [21] detected the protein expression profile of serum exosomes from healthy controls and patients with IBD to explore new IBD serological biomarkers. Exosomes were extracted from the sera of healthy controls and patients, and the proteins in exosomes were identified by label-free liquid chromatography/mass spectrometry (LC-MS/MS). A total of 633 proteins were identified. Among these proteins, PZP showed significant expression differences between patients and healthy controls, and its expression level in serum exosomes of IBD patients was higher. Furthermore, the secreted protein was related to immunosuppression, suggesting that it affected the pathogenesis of IBD by regulating immunity.

#### 2.1.3. Serum Exosomal miRNAs

CD serum exosomes induce the expression of proinflammatory cytokines and increase macrophages in vitro. The intervention of exosomes from CD with epithelial cells leads to the increase in intestinal epithelial barrier permeability. CD serum exosomes can also circulate into intestinal mucosa and significantly aggravate colitis [22]. Furthermore, CD changes the miRNA profile of exosomes. Analysis has shown the differential expression of let-7b-5p, which is identified as a potential contributor to macrophage activation and inflammatory response. Let-7b-5p mimic transmission mediated by serum exosomes significantly alleviates colitis. This provides a new perspective for the diagnosis and treatment of CD based on exosomes [23,24].

#### 2.1.4. Serum Exosomal lncRNAs

Nuclear paraspeckle assembly transcript 1 (NEAT1) is a recently discovered nuclear-restricted lncRNA, which participates in the immune response in many ways. The NEAT1-miRNA204-5p-phosphoinositide 3-kinase (PI3K)-AKT axis has been demonstrated as a potential mechanism for photodynamic therapy for treating colitis in mice [25]. NEAT1 mediates intestinal inflammation by regulating the tumor necrosis factor superfamily member 1B (TNFRSF1B) [26]. NEAT1 is highly expressed in serum samples and tissues of patients with IBD and participates in the inflammatory response by regulating the intestinal epithelial barrier. In IBD cells and mouse models, highly expressed NEAT1 participates in the inflammatory response by affecting exosome-mediated macrophage polarization. In contrast, NEAT1 downregulation inhibits the IBD inflammatory response by regulating the intestinal epithelial barrier and exosome-mediated macrophage polarization. Regulation of NEAT1 is a potential strategy for the treatment of IBD [27] (Table 1).

### 2.2. Salivary Exosomes

Exosomes are vesicles carrying non-coding RNAs that can exist in saliva. One of their functions is the long-distance transfer of various substances. This attribute determines exosomes to be used as a new biomarker for patients with IBD to assist in combating the pain of colonoscopy [28]. Zheng et al. [29] detected more than 2000 proteins in the salivary exosomes of IBD patients with IBD. By analysis, proteasome subunit alpha type 7 (PSMA7) was significantly different between patients and healthy controls, and the expression level was higher in the CD group and UC group. This secreted PSMA7 was related to the inflammatory response and was involved in the pathogenesis of IBD. The high level of PSMA7 in salivary exosomes of IBD may be a very promising biomarker for patients with IBD.

### 2.3. Milk Exosomes

Milk contains exosomes and miRNAs, which affect the pathology of intestinal inflammation. Exosomes and miRNAs are derived from endogenous synthesis and can also be supplemented by diet, such as milk [30].

Exosomes from breast milk improve colitis in dextran sulfate sodium (DSS)-induced IBD mouse models. Human milk-derived exosomes (hMD-Exo) reduce the severity of DSS-induced colitis and reduce the histopathological score and colon shortening [31]. HMD-Exo treatment also decreases the expression of IL-6, TNF-α, and TNF-β. MiRNAs highly expressed in milk, such as miR-320, miR-375, and let-7, are more abundant in the colon of hMD-Exo-treated mice than in untreated mice, and miRNAs entering the colon of mice mediate the roles of exosomes. The target genes of these miRNAs are mainly DNA methyltransferase 1 (DNMT1) and DNMT3. The therapeutic effects of hMD-Exo suggest the possibility of adding hMD-Exo as a nutrient in the enteral nutrition formula of IBD patients [32].

Compared with feeding milk with sufficient exosomes and miRNAs (ERS), feeding male *Mdr1a*^−/−^ mice with milk lacking exosomes and miRNAs (ERD) results in a 60% loss of exosomal bioactivity. The stromal collapse, gland hyperplasia, and additive microscopic disease scores of ceca in ERS mice are significantly lower than those in ERD mice, and the level of serum chemokine (C-X-C motif) ligand 9 (CXCL9) in ERS mice also has the same low index [33,34]. There are 87 differentially expressed mRNAs in the ceca of ERS and ERD mice. Among them, 16 mRNAs are related to immune function. Interestingly, miR-200a-3p is a negative regulator of the proinflammatory CXCL9, and the miR-200a-3p level is lower in livers and ceca from ERD mice than from ERS mice. The absence of milk exosomes and miRNAs aggravates the intestinal inflammation in *Mdr1a*^−/−^ mice, suggesting the importance of diet through exosomes and their inclusions in IBD pathology [35].

### 2.4. Fecal Exosomes

Autologous exosome transfer is a new personalized treatment concept which has been successfully tested in UC model mice. For this treatment, fecal exosomes are isolated from four different stages of DSS-induced mice, including pre-treatment, DSS induction, healing, and recovery. Firstly, intestinal exosomes are isolated and purified from feces by multi-step sucrose gradient ultracentrifugation [36]. Macrophages activated by LPS are used to detect the anti-inflammatory ability of fecal exosomes in vitro. In order to evaluate the anti-inflammatory activity of fecal exosomes in vivo, fecal exosomes in the healing stage of DSS-induced colitis mice are collected and fed into the digestive tract of colitis model mice by gavage to observe the in vivo effect of fecal exosomes. Evidence shows that exosomes in the healing period show the best anti-inflammatory effect in vitro and promote wound healing. Further, the model mice with oral fecal exosomes show a good effect on the prevention of UC. In the context of individualized drugs, oral autologous exosomes in the healing period may be a safe and effective method for the treatment of UC in a given patient [37,38].

## 3. Exosomes in the Pathogenesis of IBD

### 3.1. Exosomes from Human Umbilical Cord Mesenchymal Stem Cells (hucMSCs)

**Figure 2:**
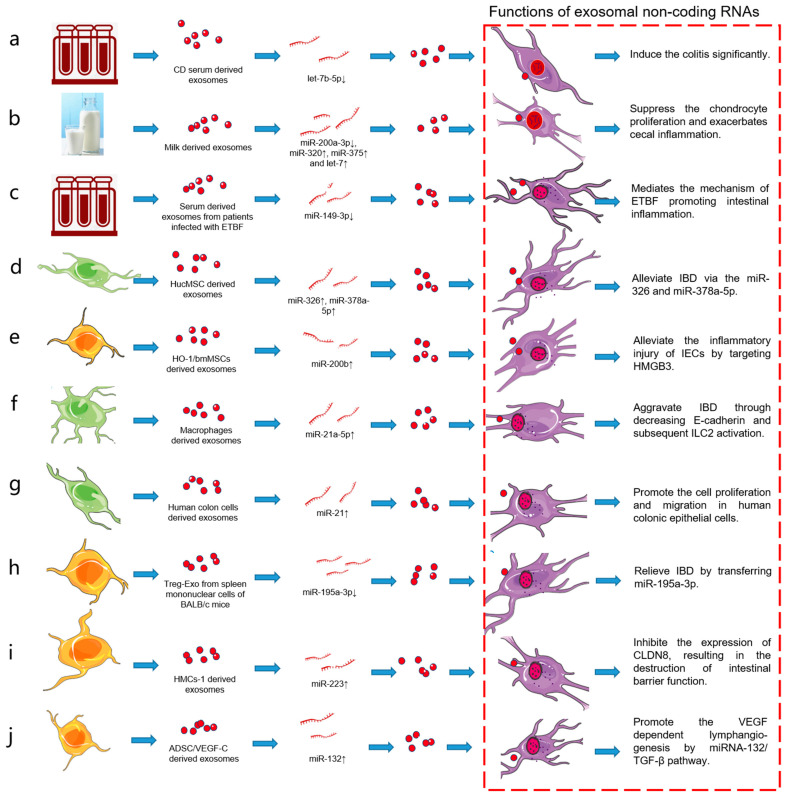
Exosomes from different sources affect the pathogenesis of IBD. Exosomes play an important role in the pathogenesis of IBD through their non-coding RNA, involving immune response disorder, barrier function, and intestinal microbiota; (**a**) CD serum-derived exosomes; (**b**) milk-derived exosomes; (**c**) serum-derived exosomes from patients infected with ETBF; (**d**) HucMSC-derived exosomes; (**e**) HO-1/bmMSCs-derived exosomes; (**f**) macrophage-derived exosomes; (**g**) human colon cells-derived exosomes; (**h**) Treg-Exo from spleen mononuclear cells of BALB/c mice; (**i**) HMCs-1-derived exosomes; (**j**) ADSC/VEGF-C-derived exosomes.

HucMSC-derived exosomes (hucMSC-exo) show therapeutic ability in DSS-induced colitis model mice by inhibiting the inflammatory mechanism. HucMSC-exo can reduce the levels of proinflammatory cytokines TNF-α, IL-1β, and IFN-γ, increase the levels of anti-inflammatory cytokines IL-10 and TGF-β1, but have no significant effect on the proliferation of peripheral blood mononuclear cells (PBMCs). HucMSC-exo significantly improves the disease activity index score, weight loss, colon shortening, and histological colitis score by inhibiting the inflammatory response, and this effect is directly proportional to the dose of hucMSC-exo [39,40] (Figure 2).

In the IBD model of BABL/C mice induced by DSS, hucMSC-exo has a certain therapeutic effect on the repair of IBD. HucMSC-exo can inhibit the process of neddylation and alleviate the symptoms of DSS-induced model mice, accompanied by the inhibition of the binding of the neural precursor cell-expressed developmentally downregulated gene 8 (NEDD8) to cullin 1. Moreover, activation of the nuclear factor-kappaB (NF-κB) signaling pathway is inhibited and the levels of neddylation-related enzyme molecules are decreased. Sequencing shows that miR-326 is highly expressed in hucMSC-exo and plays an important role in inhibiting neddylation. HucMSC-exo inhibits the neddylation through miR-326 and alleviates the DSS-induced IBD in a mouse model [41].

Macrophage pyroptosis, the cell death process after NOD-like receptor family pyrin domain-containing 3 (NLRP3) inflammasome activation, is considered to be part of the cause of an abnormal immune response in IBD pathogenesis [42]. Macrophage pyroptosis plays an important regulatory role in the reduction in colitis by hucMSC-derived exosomes. In vivo experiments have shown that hucMSC-derived exosomes inhibit the activation of NLRP3 inflammasomes in mouse colon, and the secretion of IL-1β, IL-18, and Caspase-1 cleavage is also inhibited, resulting in the reduction in cell pyroptosis. The same results are observed in in vitro cell experiments. Co-culturing of human myeloid leukemia mononuclear (THP-1) cells and mouse peritoneal macrophages (MPMs) with hucMSC-derived exosomes results in decreased expression of NLRP3 inflammasomes and increased cell survival. In addition, miR-378a-5p is highly expressed in hucMSC-derived exosomes, which play an important role in the repair of colitis with miR-378a-5p as the target [43].

The exosomes released by hucMSCs can significantly reduce the severity of DSS-induced IBD model mice. In the colon and spleen of mice treated with hucMSC-derived exosomes, the expression of IL-10 increases while the expression of TNF-α, IL-1β, IL-6, iNOS, and IL-7 decreases. The treatment of hucMSC-derived exosomes can significantly reduce the infiltration of macrophages into colon tissue in model mice. Further, the exosomes of hucMSC alleviate DSS-induced IBD by regulating the expression of IL-7 in macrophages [44].

Studies have shown that hucMSC-exo reduced the symptoms of DSS-induced IBD model mice through ubiquitination. In IBD model mice, the gene expression levels of TNF-α, IL-1β, IL-6, NEDD8 activation enzyme (NAE1), ubiquitin-conjugating enzyme E2M, and ubiquitin-like modifier activating enzyme 3 (UBA3) increased significantly, while the gene expression levels of IL-10 and interferon-induced protein 10 (IP-10) decreased significantly. The expression of these genes in the hucMSC-exo treatment group was opposite to that in the IBD model group. Compared with IBD model mice, the protein expression of K48, K63, and feather keratin 2-like (FK2) decreased significantly in the hucMSC-exo treated group, and hucMSC-exo-treated mice restored tissue structure integrity [45,46].

Exosomes secreted by hucMSCs can repair the intestinal barrier to treat IBD. TNF-α stimulated gene 6 (TSG-6) is a glycoprotein secreted by hucMSCs with tissue repair and immune regulation functions. In the IBD mouse model induced by DSS or 2,4,6-trinitrobenzenesulfonic acid (TNBS), exosomes extracted from the culture supernatant of hucMSCs are injected intraperitoneally, and intraperitoneal injection of hucMSC-exo significantly improves the IBD symptoms and reduces mortality [47]. The protective effect of hucMSC-exo on the intestinal barrier improves the destruction of tight junction structure and microvilli and increases the expression of tight junction proteins. HucMSC-exo also regulates type 2 T helper (Th2) and Th17 cell responses in mesenteric lymph nodes (MLN). In contrast, knockout of TSG-6 eliminates the therapeutic effects of hucMSC-exo on the mucosal barrier maintenance and immune regulation, while recombinant human TSG-6 (rhTSG-6) administration shows similar efficacy to hucMSC-exo [48].

### 3.2. Exosomes from Bone Marrow MSCs (bmMSCs)

Evidence shows that local injection of bmMSCs can promote the closure of perianal fistulas in CU [49]. Local injection of bmMSCs reduces experimental colitis in model mice. BmMSCs can stimulate tissue regeneration by regulating the immune response and cell–cell contact. BmMSCs also play a role through exosomes, which are membrane-encapsulated vesicles containing non-coding RNA, proteins, and lipids. Exosomes derived from bmMSCs stimulate epithelial regeneration, and the local application of exosomes as a cell-free substitute for MSC treatment reduces epithelial injury of the IBD mouse model [50].

In chemically induced colitis model mice, administration of exosomes from human bone marrow-derived MSCs (hmdMSC-Exo) significantly reduces colitis. HmdMSC-Exo can effectively reduce intestinal fibrosis, downregulate the inflammatory response, maintain intestinal barrier integrity, and polarize M2b macrophages [51]. HmdMSC-Exo mainly acts on colonic macrophages. After hmdMSC-Exo treatment, macrophages from the colon have obvious resistance to inflammatory re-stimulation. Macrophage depletion blocks the beneficial effects of hmdMSC-Exo, which further verifies the above mechanism. The non-coding RNAs and proteins rich in exosomes mediate the roles of hmdMSC-Exo, especially the non-coding RNAs, and metallothionein-2 in hmdMSC-Exo is necessary for inhibiting the inflammatory response. HmdMSC-Exo regulates the inflammatory response and may be a promising candidate target for the treatment of IBD [52].

Heme oxygen-1 (HO-1)-modified bmMSCs effectively reduce the inflammatory injury of intestinal epithelial cells (IECs) by secreting exosomes. In the co-culture system of HO-1/bmMSCs and IEC-6 cells (IEC-6s), exosomes reduced the apoptosis of IEC-6s in an inflammatory environment and reduced the expression of zona occludens 1 (ZO-1) tight junction protein [53,54]. The upregulated miR-200b in exosomes from co-cultured HO-1/bmMSCs and IEC-6s plays a role in this biological process by targeting the 3′ UTR of high mobility group box 3 (HMGB3). MiR-200b overexpression reduces the inflammatory injury of IEC-6s, while miR-200b knockout significantly blocks the protective effect of HO-1/bmMSC exosomes on the inflammatory injury of IEC-6s. In conclusion, exosomes from HO-1/bmMSCs reduce the inflammatory injury of IECs, and the mechanism is related to miR-200b targeting the hmgb3 gene in IECs induced by inflammatory injury [55,56,57].

BmMSC-exo contain a variety of proteins, mRNAs, and miRNAs, mediate a variety of biological functions, and are the main communication mechanism responsible for the communication between bmMSCs and injured cells [58]. BmMSC-exo can significantly alleviate the colitis of model mice induced by TNBS. Intravenous injection of bmMSC-exo reduces the severity of colitis, disease activity index (DAI), and colon injury [59]. Intravenous injection of bmMSC-exo significantly reduces NF-κB, TNF-α, inducible nitric oxide synthase (iNOS) and cyclooxygenase-2 (COX-2) levels, significantly inhibits the expression of IL-1β and increases the expression of IL-10. Further, BmMSC-exo can inhibit oxidative interference by inhibiting the activities of myeloperoxidase (MPO) and malondialdehyde (MDA) and upregulating superoxide dismutase (SOD) and glutathione (GSH). BmMSC-exo can also inhibit apoptosis by reducing the cleavage of caspase-3, caspase-8, and caspase-9 in colitis model rats. Therefore, the beneficial effects of bmMSCs are due to the inhibition of proinflammatory cytokine expression, blocking of the NF-κB signal transduction pathway, regulation of the antioxidant/oxidative balance, and the inhibition of apoptosis [60,61].

### 3.3. Exosomes from Olfactory Ecto-Mesenchymal Stem Cells (OE-MSCs)

OE-MSCs are a new group of resident stem cells in the olfactory lamina propria with strong immunosuppressive functions [62]. Similar to the exosomes released by bmMSCs, exosomes derived from OE-MSCs (OE-MSC-Exo) play an extension role through their non-coding RNAs and proteins. OE-MSC-Exo participate in immune regulation through their non-coding RNAs and proteins. OE-MSC-Exo have a strong inhibitory function in CD4^+^ T cell proliferation, accompanied by the decrease in IL-17 and IFN-γ secreted by T cells and the enhancement of TGF-β and IL-10 [63]. OE-MSC-Exo treatment significantly reduces the severity of this disease. After OE-MSC-Exo treatment, Tregs increase and the Th1/Th17 subsets decrease significantly. Further, OE-MSC-Exo promote the induction of Tregs and inhibit the differentiation of Th1 and Th17 cells. The function of OE-MSC-Exo in regulating T cell responses suggests that the OE-MSC-Exo may be a new target for the treatment of IBD [64].

### 3.4. Exosomes from Macrophages

Macrophages are divided into M1 and M2 subtypes according to their activation status. M1 macrophages secrete proinflammatory factors such as TNF-α, IL-1α, IL-1β, IL-6, CXCL9, and CXCL10. M2 macrophages produce anti-inflammatory mediators, including IL-10, TGF-β, C-C motif chemokine ligand 1 (CCL1), CCL17, CCL18, and CCL22 [65,66].

M2 macrophages are further subdivided into M2a, M2b, M2c, and M2d subtypes. Evidence shows that M2a, M2b, and M2c subtypes play a role in immune regulation, anti-inflammatory activity, tissue remodeling, and Th2 activation [67]. M2b macrophage-derived exosomes significantly reduce the severity of DSS-induced colitis model mice. Further, the number of Tregs and IL-4 level in the spleen of colitis model mice increases after treatment with M2b macrophage-derived exosomes. Cytokines associated with colitis inflammation (IL-1β, IL-6, and IL-17A) are significantly inhibited after treatment with M2b macrophage exosomes. The protective effects of M2b macrophage exosomes on DSS-induced colitis are mediated by the CCL1/CCR8 axis [68].

Macrophage-derived exosomes are involved in the pathogenesis of UC [69]. Exosomal miR-19b-3p communicates with tubular epithelial cells and M1 macrophages [70]. The expression of miR-21a-5p is increased in peritoneal exosomes of DSS-induced model mice. These exosomes, containing miR-21a-5p, are mainly derived from peritoneal M1 macrophages. The expression of miR-21a-5p is negatively correlated with the expression of E-cadherin in intestinal cells. Further, miR-21a-5p is involved in subsequent type 2 innate lymphoid cells (ILC2) activation. Killer-cell lectin-like receptor G1 (KLRG1), the surface inhibitory receptor of ILC2, participates in the excessive activation of ILC2 in UC by promoting the GATA binding protein 3 (GATA-3). In conclusion, exosomal miR-21a-5p from M1 macrophages aggravates the UC by reducing E-cadherin and subsequent ILC2 activation [71].

Compared with RAW264.7 macrophages treated with exosomes isolated from control mice, macrophage exosomes from DSS-induced model mice induce the phosphorylation of p38 and extracellular signal-regulated kinase (ERK) and the production of TNF-α. Proteomic analysis of RAW264.7 macrophages treated with two types of exosomes identifies 56 differentially expressed proteins, most of which are acute-phase proteins and immunoglobulins. These proteins participate in complement and coagulation cascades and are related to macrophage activation [72].

### 3.5. Exosomes from Intestinal Epithelial Cells (IECs)

In normal conditions, IECs constitutively stimulate CD4^+^ Tregs. However, in the pathogenesis of IBD, the major histocompatibility complex (MHC) II-restricted antigen presentation is involved in the stimulation of proinflammatory CD4^+^ T cells [73].

Gasdermin D (GSDMD) induces pyroptosis through the pore-forming activity of its N-terminal domain, which is cleaved by the activated caspase related to IL-1β release [74]. Increased expression of epithelial GSDMD is observed in patients with IBD and experimental colitis model animals, and the epithelial-derived GSDMD mediates the release of insoluble IL-1β during experimental colitis [75]. In cells, GSDMD and IL-1β are co-located with exosomal markers CD63 and Alix. Accompanied by Cdc37/Hsp90, the GSDMD recruits E3 ligase neuronally expressed developmentally downregulated 4 (NEDD4) to catalyze the polyubiquitination of pro-IL-1β as a signal for cargo loading into secretory vesicles. GSDMD and NEDD4 are necessary for the release of CD63^+^ small extracellular vesicles (sEVs) containing IL-1β, GSDMD, NEDD4, and caspase-8 from IECs. GSDMD-dependent release of IL-1β-containing sEVs can be detected in cultured colonic explants of colitis model mice, and GSDMD deficiency significantly reduces the severity of the disease, indicating that GSDMD-mediated sEV release plays an important role in the pathogenesis of intestinal inflammation [76].

### 3.6. Exosomes from Colonic Epithelial Cells (CECs)

Substance P (SP) is a neuropeptide/hormone, and its high-affinity receptor neurokinin type 1 (NK-1R) is highly expressed in the process of colitis. SP/NK-1R signal stimulates the differential expression of miRNAs and promotes the proliferation of CECs [77]. MiR-31-3p is involved in SP-associated inflammation in human CECs and experimental colitis model mice [78]. Further, SP/NK-1R signaling in human CECs (in vitro) and mouse colon (in vivo) promotes the production of exosomes and upregulates the level of miR-21 in exosomes. SP/NK-1R signaling regulates the biogenesis of exosomes and induces miR-21 cargo sorting. Cell proliferation and migration induced by SP/NK-1R depend at least in part on intercellular communication through exosomal miR-21. Furthermore, miR-21, derived from exosomes induced by SP stimulation, promotes the proliferation and migration of human colon cells and cells in mouse colonic crypts [79].

Interestingly, the establishment of the IBD model will lead to significant barrier destruction through the increase in transepithelial-electrical resistance (TER), permeability-coefficient (Papp), sICAM sE-selectin, and IL-8 [80]. Prospective drug–vehicle (silica nanoparticles, aSNP) models affect persistent inflammation by affecting the above factors. This is due to the observed decrease in soluble intercellular adhesion molecule (sICAM)/sE-selectin after aSNP exposure to inflammatory endothelium, which is related to the decrease in the secretion of exosomes containing ICAM/E-selectin. Exosome-mediated intercellular communication is not only affected by exosome secretion, but also by intestinal mucosal barrier [81,82].

### 3.7. Exosomes from Dendritic Cells (DCs)

The exosomes of DCs secreting TGF-β1 (sTGF-β1-exo) delay the development of IBD model mice, and the exosomes from DCs expressing membrane-associated TGF-β1 (mTGF-β1-exo) have stronger immunosuppressive activity than sTGF-β1-exo in vitro [83]. For example, mTGF-β1-exo treatment of model mice inhibits the progression of EAE induced by myelin oligodendrocyte glycoprotein (MOG), even after disease onset. MTGF-β1-exo treatment maintains the regulatory capacity of CD4^+^ Foxp3^+^ Tregs [84].

Moreover, Tregs of EAE model mice treated with mTGF-β1-exo significantly prevent the development of EAE in the receptor. Furthermore, mTGF-β1-exo treatment also impairs Ag-specific Th1 and IL-17 responses in model mice, but promotes the expression of IL-10, which is related to the activation of p38, ERK, Stat3, and NF-κB and the downregulation of IL-6 expression in DCs. In view of the strong immunosuppressive ability of mTGF-β1-exo, these exosomes may be a potential therapeutic target for IBD [85,86].

Exosomes secreted by DCs have an effect on IBD pathology. DCs-derived exosomes regulate the immune response and prevent the development of autoimmune diseases [87]. Interestingly, DC exosomes treated with soluble eggs antigen (SEA) of *Schistosoma japonicum* contribute to the treatment of IBD. Exosomes are purified from the supernatant of DCs treated with or without SEA. After administration of SEA-treated DC exosomes, a significant decrease in weight loss and disease activity index of DSS-induced colitis model mice is observed; moreover, colon length is improved and the mean colon macroscopic score is reduced. In addition, SEA-treated DC exosomes can prevent colon injury in DSS-induced acute colitis mice. In conclusion, SEA-treated DC exosomes are more effective in reducing the severity of DSS-induced acute colitis in mice than DC exosomes without SEA [88].

IL-10 plays an important role in the development of normal mucosal immunity. The exosomes derived from IL-10-treated bone marrow DCs reduce the incidence rate and severity of collagen-induced arthritis (CIA) model mice [89]. For TNBS-induced colitis model rats, intraperitoneal injection of IL-10 exosomes significantly reduced all clinical and histopathological parameters. The therapeutic effects of IL-10 exosomes are related to the downregulation of mRNA expression of IL-2, IFN-γ, and TNF-α in colon tissue. Further, IL-10 exosome treatment results in significant upregulation of IL-10 mRNA expression in colonic tissue and Tregs in colonic lamina propria. The good therapeutic effects of IL-10 exosomes provide a promising new strategy for the treatment of IBD [90].

### 3.8. Exosomes from Tregs

Exosomes are a new mechanism for Tregs to play biological roles, that is, Tregs play a therapeutic role in IBD by secreting exosomes (Treg-Exo). For example, isolated Treg-Exo from spleen mononuclear cells of BALB/c mice were injected into IBD model mice induced by DSS to observe the therapeutic effects of Treg-Exo on IBD. Data showed that Treg-Exo can reduce the DSS-induced IBD model mice. In the presence of TNF-α, the co-culture model of Treg-Exo and colonic epithelial YAMC cells is used to study the communication between Tregs and IECs. The results showed that Treg-Exo can be transferred to YAMC cells. For YAMC cells, Treg-Exo promote cell proliferation and inhibit apoptosis. When the expression of miR-195a-3p in Treg-Exo is inhibited, the above therapeutic effects of Treg-Exo are eliminated. Further, miR-195a-3p plays a therapeutic role with pro-apoptotic Caspase 12 as a direct target [91,92].

### 3.9. Exosomes from Granulocytic Myeloid-Derived Suppressor Cells (G-MDSCs)

Exosomes released by G-MDSCs alleviate the DSS-induced colitis model mice. Mice treated with G-MDSCs-derived exosomes (G-MDSC-exo) show stronger resistance to colitis, which is reflected in the decrease in disease activity index and inflammatory cell infiltration [93]. In model mice treated with G-MDSC-exo, the levels of serum IFN-γ and TNF-α decrease significantly. Furthermore, G-MDSC-exo treatment can significantly reduce the proportion of Th1 cells in MLNs of colitis model mice and increase the proportion of Tregs. Interestingly, the knockout of arginase (Arg)-1 in G-MDSC-exo eliminates the spontaneous improvement of colitis model mice, suggesting that G-MDSC-exo play a role by targeting the Arg-1. In addition, G-MDSC-exo can inhibit CD4^+^ T cell proliferation and IFN-γ secretion in vitro and inhibit the delayed-type hypersensitivity (DTH), which is also related to Arg-1 activity. These findings suggest that G-MDSC-exo alleviate the DSS-induced colitis model mice by inhibiting Th1 cell proliferation and promoting Tregs expansion [94,95].

### 3.10. Exosomes from Human Mast Cells-1 (HMCs-1)

HMCs-1-derived exosomes significantly upregulate intestinal epithelial permeability and destroy intestinal barrier function, which is attributed to exosome-mediated transfer of functional miRNAs from HMCs-1 [96]. These transferred miRNAs further lead to the inhibition of tight junction-related proteins expression, including Claudin 8 (CLDN8), tight junction proteins 1 (TJP1), and Occludin (OCLN). HMCs-1-derived exosomes are rich in miR-223 and can inhibit CLDN8 expression. MiR-223 inhibitors significantly reverse the inhibitory effect of HMCs-1-derived exosomes on CLDN8 expression, which further verifies the important role of miR-223. Obviously, exogenous miR-223 enriched in HMCs-1-derived exosomes inhibits the expression of CLDN8, resulting in the destruction of intestinal barrier function. HMCs-1-derived exosomes and miR-223 may be new therapeutic targets for IBD [97,98].

### 3.11. Exosomes from Adipose-Derived MSCs

Exosomes secreted by adipose-derived MSCs (adMSC-exo) affect the pathological changes of IBD. In DSS-induced colitis, intraperitoneal injection of adMSC-exo reduces colonic bleeding, colonic shortening, colonic injury, and weight loss [99]. Intraperitoneal injection of adMSC-exo decreases TNF-α, IFN-γ, IL-12, and IL-17 levels, and upregulates IL-4, TGF-β, and IL-10 levels in the lymph nodes and spleen. Further, intraperitoneal injection of adMSC-exo also increases the percentages of CD4^+^ CD25^+^ Foxp3^+^ Treg cells in the lymph nodes and spleen of mice. Therefore, adMSC-exo can regulate the Treg population percentage and improve inflammation in DSS-induced colitis [100].

Vascular endothelial growth factor-C (VEGF-C) is a powerful lymphangiogenic factor, and lymphangiogenesis plays an important role in the pathogenesis of IBD [101]. In exosomes isolated from VEGF-C-treated adipose-derived stem cells (ADSCs) (ADSC/VEGF-C), the level of miR-132 was significantly higher than that isolated from ADSCs control, and the highly expressed miR-132 migrated from ADSCs to lymphatic endothelial cells (LECs) through exosomes. Exosomes from ADSCs/VEGF-C more effectively promoted LEC proliferation, migration, and tube formation through miR-132 than exosomes from ADSCs. The treatment of ADSCs with miR-132 inhibitors can weaken VEGF-C-dependent lymphangiogenesis, reflecting the importance of miR-132 for ADSC/VEGF-C exosomes. Further, miR-132 promotes the lymphangiogenic response by directly targeting Smad-7 and regulating TGF-β/Smad signaling. This indicates that exosome-mediated miRNAs have an important effect on lymphangiogenesis in the pathological mechanism of IBD [102,103].

## 4. Exosomes Derived from Pathogenic Microbial Infection

### 4.1. Exosomes and Helicobacter Pylori (Hp) Infection

Hp infection has a protective effect on IBD through serum exosomes. Chen et al. [104] treated human IECs with serum exosomes from patients with HP-positive chronic gastritis (Hpcg-Exo) and detected the expression of cytokines and signal pathway genes with an antibody microarray or PCR array. The results showed that the Hpcg-Exo promoted the expression of NLRP12 in IECs, and the upregulated NLRP12 decreased the expression of chemokines MIP-1α and MCP-1 by inhibiting the Notch signaling pathway. Hpcg-Exo attenuated the inflammatory response of DSS-induced colitis model mice and improved the symptoms of colitis. NLRP12 knockout reversed the effects of Hpcg-Exo, proving that the anti-IBD effects of Hpcg-Exo in vivo are related to NLRP12. Clinical sample studies showed that NLRP12 negatively correlated with disease activity in children with IBD. These findings provide a new theoretical basis for the protective mechanism of HP infection in IBD [105,106].

### 4.2. Exosomes and Enterobacter Infection

A high prevalence of adherent-invasive *Escherichia coli* (AIEC) has been reported in the intestinal mucosa of patients with CD. Exosomes released from human IECs infected with CD-associated AIEC activate host innate immune responses and enhance bacterial intracellular replication [107].

From healthy controls to patients with IBD, the level of serum exosomal miR-149-3p decreases significantly. MiR-149-3p in serum exosomes is negatively correlated with Enterotoxigenic Bacteroides fragilis (ETBF) abundance in IBD, and ETBF reduces miR-149-3p expression which depends on METTL14-mediated m6A methylation, resulting in the promotion of the pathological development of IBD. As a target gene of miR-149-3p, PHF5A transactivates SOD2 by regulating KAT2A mRNA alternative splicing. Therefore, targeting the ETBF/miR-149-3p pathway is a promising method for the treatment of IBD [108,109].

### 4.3. Exosomes and the Infection of Gastrointestinal Parasites

Gastrointestinal parasites such as hookworm have evolved to cause minimal damage to the host in order to establish a chronic infection. In fact, hookworm has a powerful inflammatory inhibitory function, which has been used in the experimental study of treating IBD, and some of its immune inhibitory functions have been realized through the exosome pathway [110]. For example, exosomes of the rodent parasite *Nippostrongylus brasiliensis* (*N. brasiliensis*) can drive the parasitism of *N. brasiliensis* after being actively internalized by mouse intestinal organs. There are 81 kinds of proteins and 27 kinds of sperm-coating protein-like extracellular proteins in the exosomes secreted by *N. brasiliensis*. There are also 52 kinds of miRNAs in the exosomes secreted by *N. brasiliensis*, many of which are involved in inflammatory regulation [111]. The intestines of mice receiving a single intraperitoneal injection of exosomes show protection from colitis inflammation. Cytokines related to colitis pathology (such as IL-6, IL-1β, IFNγ, and IL-17a) are significantly inhibited in the colon tissue of exosome-treated model mice. In contrast, the expression of anti-inflammatory cytokine IL-10 is significantly increased in *N. brasiliensis* exosomes-treated mice. Moreover, proteins and miRNAs contained in *N. brasiliensis* have great potential applications in the treatment of worm infection and IBD [112,113].

## 5. Conclusions and Research Prospects

This work systematically reviewed the latest research progress for exosomes in IBD and the feasibility of the use of exosomes in body fluids as diagnostic targets for IBD, especially the roles of exosomes as non-coding RNA and protein delivery systems in the pathogenesis of IBD. The mechanisms of exosome intervention in IBD pathogenesis involve the immune response, barrier function, and intestinal flora. Exosomes in body fluids, including serum exosomes, salivary exosomes, and milk exosomes, can be used as new targets for the diagnosis and treatment of IBD, which is of great significance for the early diagnosis and treatment of patients with IBD. Further, exosomes from various cells play an important role in the pathogenesis of IBD and affect the occurrence and development of this disease. In IBD, hucMSCs, bmMSCs, OE-MSCs, macrophages, IECs, CECs, DCs, Tregs, G-MDSCs, HMC-1, and adipose-derived MSCs all secrete exosomes and intervene in the pathological mechanisms of IBD. Interestingly, Hp infection, enterobacter infection, and gastrointestinal parasites-mediated exosomes also affect the pathological changes of IBD, which explains the relationship between pathogenic microbial infection and IBD pathogenesis. This review helps to clarify the pathogenesis of IBD and provides a new perspective for IBD to explore new diagnostic markers and therapeutic targets.

In the future, exosomes and IBD research should include the following three aspects. First, exosomes deliver non-coding RNAs and proteins to participate in the information exchange between stem cells and damaged cells, which have significance for the development of new exosome-based drug delivery systems [114]. Based on this design, grape exosome-like nanovesicles (GELNs) can be used as oral drug delivery vehicles because of their inherent biocompatibility and biodegradability. In patients with IBD, intestinal stem cells are difficult to obtain by common exogenous drugs in vivo, and the oral Syrah GELN provides a new way to regulate the microenvironment of stem cells to promote intestinal remodeling. This may be a new way to treat IBD in the future [115,116]. In the future, we can design more biocompatible nanovesicles as carriers for in vivo drug delivery, which is also a research hotspot in the field of pharmacy. However, how to deliver drug-loaded nanovesicles to the lesion site accurately and bind to the corresponding antigen is still a challenge.

Second, chronic inflammation, which is difficult to cure, can induce cancer, and there is a certain correlation between inflammation and cancer [117]. Exosomes may be involved in this pathological mechanism. Colonic exosomes affect fibroblast proliferation, and colonic exosomes mediate the transformation of IBD to tumors. In addition to participating in the pathogenesis of IBD, exosomes are related to cancer pathology, including uncontrolled tumor growth and metastasis. According to current research findings, the roles of exosomes are mediated by non-coding RNAs and proteins contained therein. For example, comparing the protein expression profile of colorectal exosomes isolated from healthy mice and DSS-induced colitis model mice, model mice-derived exosomes significantly promoted cell proliferation in an epidermal growth factor receptor (EGFR)-dependent manner. Inflamed colon-derived exosomes promoted the transformation of inflamed colon to colon tumor by activating fibroblasts [118,119]. The study of IBD exosomes involved in the transformation of intestinal inflammation into colon cancer has important reference value for the transformation of inflammation into other cancers. This is a new research direction of cancer pathogenesis for future study.

Third, the relationship between milk exosomes and IBD pathology reveals the milk–exosome–intestine-IBD axis relationship, which has important reference value for the treatment of diseases with a milk diet. Intestinal inflammation, imbalance of intestinal microbiota, and enhanced intestinal leakage are not only related to intestinal diseases but also to the characteristics of many other systemic inflammatory diseases, such as systemic lupus erythematosus (SLE), rheumatoid arthritis (RA), and systemic sclerosis [120]. Milk-derived exosomes may be a potential therapeutic strategy with which to regulate intestinal inflammation. Since the impact of milk-derived exosomes on human health depends on the type of dairy products, personal health status, and personal preference for dairy products, several aspects need to be considered before such clinical trials: (1) separating pure cellular exosomes without other milk components, to better evaluate the separate effects of exosomes and eliminate the interference of milk antigens; (2) systematically studying the miRNAs, proteins, and lipids contained in milk exosomes to clarify the mechanisms of exosomes; (3) carrying out systematic research on exosome toxicology and side effects to ensure the safety of milk-derived exosomes.

## Figures and Tables

**Table 1:** Exosomes and inclusions reported in IBD.

Disease	Classification of Exosomes	Non-Coding RNAs	Expression in Target Cells	Regulatory Roles	Targets	Reference
IBD (CD)	CD serum-derived exosomes	let-7b-5p	Decreased in CD serum-derived exosomes	Let-7b-5p mimic delivery alleviates colitis significantly.	TLR4 pathway	[24,25,26,27,28,29,30,31]
IBD	Milk-derived exosomes	miR-320, miR-375 and let-7	Increased in the colon of model mice	Suppress the chondrocyte proliferation and migration	DNMT1, DNMT3	[32,33,34]
IBD	Milk-derived exosomes	miR-200a-3p	Decreased in livers and ceca from ERD mice	Milk exosome and miRNA depletion exacerbates cecal inflammation in *Mdr1a*^−/−^ mice.	CXCL9	[35,36,37,38,39,40]
IBD	HucMSC-derived exosomes	miR-326	Increased in hucMSCs-derived exosomes	HucMSC-exo inhibits the neddylation through miR-326 and alleviates the DSS-induced mouse model.	NF-κB signaling pathway, neddylation-related enzymes	[41,42]
IBD	HucMSCs-derived exosomes	miR-378a-5p	Increased in hucMSCs-derived exosomes	Attenuate colitis by regulating macrophage pyroptosis via the miR-378a-5p/NLRP3 axis	NLRP3, IL-1β, IL-18, and Caspase-1	[43]
IBD	HucMSC-derived exosomes	-	-	Protected against IBD through restoring mucosal barrier repair and intestinal immune homeostasis via TSG-6 in mice.	TSG-6	[44,45,46,47,48,49,50,51]
IBD	HmdMSC-derived exosomes	-	-	HmdMSC-Exos reduce murine colonic inflammation via a macrophage-dependent mechanism.	Non-coding RNAs and metallothionein-2	[52,53,54]
IBD	HO-1/bmMSCs-derived exosomes	miR-200b	Increased in exosomes co-cultured with HO-1/BMMSCs and IEC-6s	Alleviates inflammatory injury of IECs by targeting HMGB3.	HMGB3	[55]
IBD	Macrophage-derived exosomes	-	-	Exosomes derived from M2b macrophages attenuate DSS-induced colitis.	CC chemokine 1 (CCL1)/CCR8 axis	[56,57,58,59,60,61,62,63,64,65,66,67,68,69,70]
IBD	Macrophage-derived exosomes	miR-21a-5p	Increased in peritoneal exosomes of model mice	Aggravates IBD through decreasing E-cadherin and subsequent ILC2 activation.	E-cadherin and ILC2	[71,72,73,74,75]
IBD	Human colon cell-derived exosomes	miR-21	Increased in exosomes induced by SP stimulation	Promotes cell proliferation and migration in human colonic epithelial cells.	Proliferation and migration genes	[76,77,78,79]
IBD	Treg-Exo from spleen mononuclear cells of BALB/c mice	miR-195a-3p	Decreased in Treg-Exo from spleen mononuclear cells	Exosomes derived from Treg cells relieve IBD by transferring miR-195a-3p.	Caspase 12	[80,81,82,83,84,85,86,87,88,89,90,91,92,93,94]
IBD	MDSC-derived exosomes	Arg-1	Increased in G-MDSC-exo	Attenuates the DSS-induced colitis in model mice.	IFN-γ and TNF-α	[95,96,97]
IBD	HMCs-1-derived exosomes	miR-223	Increased in HMCs-1 derived exosomes	HMCs-1 derived exosomes inhibit the expression of CLDN8, resulting in the destruction of intestinal barrier function.	CLDN8	[98,99,100]
IBD	ADSC/VEGF-C-derived exosomes	miR-132	Increased in exosomes from ADSC/VEGF-C	Promotes VEGF-C-dependent lymphangiogenesis by regulating miRNA-132/TGF-β pathway	Smad-7 and TGF-β/Smad signaling	[101,102,103]
IBD	Hpcg-derived exosomes	NLRP12	Increased in intestinal epithelial cells and model mice	Hpcg derived exosomes inhibit MCP-1 and MIP-1α expression via NLRP12-Notch pathway and improve DSS-induced colitis in mice	MCP-1 and MIP-1α	[104]
IBD	Serum-derived exosomes from patients infected with ETBF	miR-149-3p	Decreased in serum exosomes	Exosomal miR-149-3p mediates the mechanism of ETBF promoting intestinal inflammation.	PHF5A	[105,106,107,108,109]

## Data Availability

The study did not report any data.
